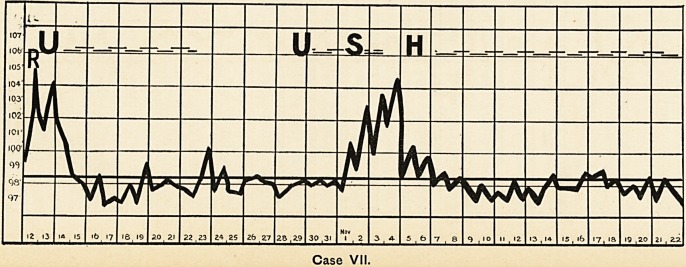# Infection of the Urinary Tract with Bacillus Coli
1Read at a Meeting of the Bristol Medico-Chirurgical Society, January 12th, 1910.


**Published:** 1910-03

**Authors:** J. R. Charles

**Affiliations:** Physician to the Bristol Royal Infirmary.


### INFECTION OF THE URINARY TRACT WITH BACILLUS COLI.[1]

BY

J. R. Charles, M.A., M.D. Cantab., M.R.C.P.,
*Physician to the Bristol Royal Infirmary.*

---

Infection of the urinary tract with B. Coli gives rise from time to time to both puzzling and alarming symptoms. In some of the cases the constitutional symptoms are very marked, when there is little to draw special attention to the urinary organs.

The infection is more common in females than males, and the onset of the symptoms not infrequently accompanied by a rigor, which may be repeated. There may be pain, in some cases urethral, in others vesical, in others lumbar, or perhaps even more frequently subcostal. There may be tenderness over the kidneys, which may be felt to be enlarged.

The urine is as a rule acid (for B. Coli does not break up urea), containing a trace or more of albumen; more rarely blood is present, and in very exceptional cases a profuse hæmaturia. Pus may be present in considerable quantities with little constitutional disturbance, while on the other hand there may be very marked symptoms when the pus is represented by a few pus cells found after centrifugalisation.

Kidneys which have been seen in this condition have been found enlarged and congested, with prominent stellate veins, and at times with subcapsular hemorrhages. Sometimes numerous yellow nodules, which are virtually minute abscesses, have been observed in the cortex. Submucous hemorrhages are sometimes seen in the renal pelvis. A diffuse degeneration of the renal epithelium has been found in the more severe cases, and an exudation of leucocytes into wedge-shaped areas in the cortex, into the glomeruli, tubes and stroma. The route by

[1] Read at a Meeting of the Bristol Medico-Chirurgical Society, January 12th, 1910.

which the organism reaches the tract is at present undecided. There is no evidence that it occurs via the lymphatic system, and therefore it is either by the blood stream or by an ascending infection.

Points in favour of a blood infection are that similar renal changes may be produced experimentally in animals if their kidneys have been injured by trauma, injection of bismuth into the pelvis, ligation of the ureter, and so on, after the organisms have been injected into the blood stream. In some cases the bladder and ureteric openings have appeared perfectly healthy on cystoscopic examination, a fact which has been used by some as an argument in favour of a blood infection as against an ascending infection. It is, however, very doubtful if this argument is valid.

On the other hand, there are several considerations which are in favour of an ascending infection. The complaint is much more frequent in women than in men, a fact which is accounted for by the greater proximity of urethra to anus and the shortness of the urethra. Secondly, when the ureters are transplanted into the rectum, an ascending infection with B. Coli not infrequently takes place in the kidneys. Moreover, there is undoubtedly in many of these cases a preliminary inflammation of the bladder, and in many cases the renal disease is unilateral. Furthermore, removal of the diseased kidney has been followed by the immediate cessation of symptoms of constitutional poisoning.

With reference to treatment, little good seems to have followed measures directed against constipation and intestinal absorption, such as the administration of salol, intestinal antiseptics, calomel and castor oil. Urotropin and helmitol seem to be regarded by several writers as of little value. This has not been in accordance with my experience, for the signs and symptoms have been much ameliorated or cured after the administration of urotropin. Those who find this class of drug of no value recommend sod. citrate, with the idea of rendering the urine alkaline, and thus inhibiting the growth of the organism.

An anticolon serum has been used, apparently with some success. It was employed in one of the cases to be presently described, but in conjunction with other measures, so that one cannot say how much good was derived from it.

Lastly, there is vaccine treatment, which is said by Dudgeon to be followed by temporary improvement, but very apt to be followed by relapses.

The following seven cases which I have seen in the last two years give illustrations of the complaint.

The first case was a lady, aged 47 (Mrs. H. M.), seen in consultation for rigors and intermittent fever, on October 21st, 1907. There was nothing in her previous history up to three years before her illness which would throw any light on the causation of the attacks. At that time she suffered from severe menorrhagia, due to a fibroid, for which she underwent panhysterectomy. Following this she had some slight phlebitis in left leg. No chance of malaria. She had always been bilious, but not jaundiced.

Her illness began in July, 1907, when she went to the Oxford pageant and felt shivery. The following day she went to Henley, and stayed a week. There she had some retching and felt generally slack. She regained her health till the middle of August, when, preceding the sale of her home, she had to sort a good many papers in a damp strong-room. She then had an attack of nausea, some vomiting and a rigor, pains in her legs and in her left iliac fossa. She was very ill for three days. The attack lasted a week, and was accompanied by frequent micturition, nothing abnormal, however, being found in the urine.

On September 20th she had a third attack, ushered in with a rigor, nausea and vomiting, marked elevation of temperature, and weak cardiac action. She thought she was going to die. This attack lasted two days. She was kept in bed for a week, and then got up for five days, when early in October she had her fourth attack, similar in character, and lasting three days. She was kept in bed two weeks, during the last ten days of which temperature and pulse were normal. On October 16th she had her fifth attack, again with a rigor and temperature of 102. On October 17th she was examined by Dr. Tate, who had removed her uterus, but who found nothing abnormal in the pelvis.

On October 21st nothing abnormal could be detected in lungs, heart, liver or spleen. There was some tenderness and rigidity over the right kidney, which was said never to have been present before. The blood has been recently examined at Easte's Laboratory, and from there a report had been sent that there was no indication of pus in the body. Examination of further stained films was negative. The urine contained a trace of

Case I.

Case IV.

R = Rigor.

U = Urotropin.

H = Helmitol.

S = Sodium Citrate.

The strokes —‒—‒— after the letters indicate the period of administration of the drugs.

Case V.

Case VII.

albumen, some pus, no casts, bladder epithelium, no T. B. A report from St. Bartholomew's Hospital, where another specimen of urine had been sent, affirmed the presence of B. Coli. The patient was put on urotropin (gr. x t.d.s.), and made an uninterrupted recovery without further relapses.

The next case (M. B.), a medical man, seen in consultation on March 24th, 1909, with Mr. Carwardine, Dr. Powell and Prof. Walker Hall, had been ill some weeks with indefinite pains about his body. Ten days previously his temperature was 101, and on March 18th he complained of frequent micturition, much pain in the bladder after the act, and a little hæmaturia. The urine was acid, contained a good deal of pus, no crystals, no casts, not more albumen than would be accounted for by the pus. An almost pure growth of B. Coli was obtained from the urine. On March 23rd he had a severe rigor, and another on March 24th. For thirty-six hours he had been troubled with excessive retching and vomiting.

On examination, the tongue was moist and clean. Nothing abnormal was found in the heart, lungs or liver. In this case the spleen could be just felt. He had an area of tenderness over the left kidney and in left loin. His retching was so severe that rectal feeding was necessary. One phial of anti-B. Coli serum was given per rectum and urotropin (gr. x t. d.s.). By March 26th he had improved ; the temperature was down. His nausea had been very much better for twenty-four hours, and he could now retain albumen water by the mouth. Prof. Walker Hall reported fewer B. Coli in the urine and the albumen less. No casts, oxalates or urates. Later, the albumen entirely cleared up. He is now quite well, and has had no relapse.

The third case was a nurse (Miss H.), seen in consultation, suffering from pain over the left renal region. Her father and mother had both died from phthisis. She had had no previous illnesses of importance. She said that in August, 1908, she had a chill, and had not been well since, being subject to pain in the epigastrium, which was worse after food ; had some slight vomiting, but never hæmatemesis or melæna. When first seen by her doctor three weeks before I saw her her symptoms were regarded as dyspeptic, and she was given bismuth and HCN, etc., without relief. For some little time she had suffered from puffiness under the eyes in the morning. Her temperature had never been found abnormal, and she had not had any rigors. On further questioning, her pain appeared to be of two varieties—(1) an acute epigastric pain and (2) a dull, aching pain in the left loin, with occasional exacerbations, sufficient to make her stop anything she might be doing, inducing sweating and nausea. The urine had from time to time been high coloured, but it was doubtful if it had contained blood. She had been losing flesh, and had suffered from very marked cold night sweats. When examined nothing abnormal was found in lungs, heart, fundi, liver, spleen or spine. There was very marked tenderness over the left kidney with rigidity. A tentative diagnosis of possible tuberculosis of the kidney was made, and she was admitted into the Bristol Royal Infirmary for further observation. There it was seen that she had a temperature of an oscillating variety; marked tenderness over the left kidney persisted. The urine was passed in about normal amounts, was acid, contained a trace of albumen, no blood, no casts, and on centrifugalisation a few pus cells were found. No T. B. were discovered. A guinea-pig was inoculated for evidence of T. B., but died a few days later, a marked growth of B. Coli occurring at the seat of injection. A few days later B. Coli were found in the urine of the patient. She was treated with urotropin; her temperature became normal, her pain disappeared, and she was sent out cured. Since that time she has had a few slight returns of her trouble, but not severe enough to interfere in any way with her work.

The fourth case was a woman (M. D.), aged 23, admitted to the Bristol Royal Infirmary on the fourth day of her illness, which began with a sharp pain in the right side, like a knife, whenever she breathed. The same afternoon she had an attack of shivering; no vomiting, and very little cough. On admission she did not look ill, and had no distress. T. 103.4, P. 112, R. 20. Heart normal. Slight diminution of entry of air at the right base. The spleen descended a quarter of an inch below the costal margin. There was no tenderness over the kidneys, though the right was movable and easily palpable. Widal was negative. The urine was acid, 1020, contained a thick cloud of albumen, no blood, no casts, but some pus. A few days later B. Coli were grown from the urine, and the patient was put on urotropin. The temperature immediately began to fall, became normal three days later, and did not rise again. The pus gradually diminished in amount, so that there was no deposit when she went out a month later; the albumen also steadily diminished, though a faint trace remained to the end. This patient was also treated by bladder washing.

The fifth case was a man (T. G.), aged 64, who came to my out-patient department complaining of dyspeptic symptoms. There was a slightly tender spot in the epigastrium, and his temperature being 101, he was admitted. For two years he had suffered from attacks similar to the present one. For five weeks he had suffered from pain in the stomach day and night. He had vomited several times, sometimes after food and sometimes when out of doors between meals. The vomiting had relieved pain. The bowels had been very costive; had never passed any blood; had lost 1½ stones during the last two years, and had never drunk to excess. Nothing of importance was found in lungs or heart. The edge of the liver could be felt just below the ribs, and there was some resistance in the upper part of the abdomen. After a test meal there was no free HCl in the stomach contents, lactic acid being present in small quantity. No mass was felt even after distending the stomach. A tentative diagnosis of gastric carcinoma was made. The urine was acid and contained no albumen or sugar. A fortnight later the patient had a series of rigors, one or two a day for five days, with a very markedly swinging temperature, which reached on one occasion 106. No evidence of any focus of pus could be found. There were 30,000 white cells, of which

91 per cent. were polymorphonuclears.
1 per cent. were small lymphocytes.
6.5 per cent. were large mononuclears.
1.5 per cent. were transitionals.

A blood culture was negative. The patient was not extremely ill between the rigors.

The type of the temperature, the recurrence of the rigors, without any cause which we could find, suggested that here again the causal factor might be a B. Coli infection of urine. Professor Walker Hall's report on this point stated that there are "gas-producing organisms belonging to the coli group, and in addition cocci, and non-fermenting rods."

The patient was put on urotropin, after which the temperature fell and he had no more rigors. In this case there was only a slight trace of albumen in the urine, and that during the height of the fever, and no pus cells to be found until a few days later, when blood also appeared. The tenderness in the epigastrium continued, and later a nodular tumour could be felt in the liver. The patient lost weight, eventually became jaundiced, and died two and a half months later, the cause of death being carcinoma of the pancreas, with secondary growths in liver and omentum. The following is the *post-mortem* report :—

"The kidney shows advanced parenchymatous changes. The capsule is slightly thickened, and there are a few minute patches of interstitial proliferation. The glomeruli are congested and swollen, and there is a slight glomerular exudate of the serous type. The proximal and distal convoluted tubules contain some bile-stained casts, and the lining cells are in a condition of late catarrhal change. The medullary tubules do not show much change, and the lining of the pelvis, although composed of more fibrous tissue than usual, does not show any defined reaction to bacterial irritation."

The sixth case is one which I saw in consultation on July 6th, 1909, a man (Mr. H.) aged 42. He had previously enjoyed good health, with the exception of a doubtful attack of rheumatism in childhood. He had been doing his work as usual till June 24th, when he felt rather seedy. For the next two days he had some diarrhœa, but this was probably due to a purgative. During this time his temperature was raised, and the notes say that from this time onward till July 6th his temperature was swinging very markedly. On June 28th he had a rigor, and other rigors followed on June 29th and July 4th, on which latter date the temperature reached 105.5. There was no enlargement of the spleen, no spots. Widal's reaction was negative. The urine contained a very faint haze of albumen ; no vomiting, no headache, but vague, diffuse pains over body.

When I saw him he was perspiring profusely ; he was restless and anxious. No definite abnormal physical signs were found in the lungs ; the heart was slightly enlarged to the left, and at the apex was a systolic murmur which was slightly conducted outwards. Nothing wrong was found with throat, ears, sinuses, joints, bones, glands, spleen, liver, fundi or nervous system. There was diffuse tenderness over the right renal region, and the urine contained a trace of albumen. The case appeared to be one of septicæmia with involvement of the mitral valve—malignant endocarditis. The urine was sent to the University for bacteriological investigation, and a report came back saying that a pure growth of an organism had been separated which gave the reactions of B. Coli.

On July 9th he was put on urotropin (gr. x 6tis. horis), which he continued for five days. Daily rigors continued. On July 14th he had a severe syncopal attack, and became slightly jaundiced with some bile in the urine. I am told that no fresh abnormal physical signs appeared. He died on the 17th, and unfortunately the chance of obtaining an autopsy was out of the question.

The last case is one of a woman, aged 24, who came up to my out-patient department and was admitted under the care of Dr. Prowse, who has very kindly permitted me to make use of his notes. She came up for cloudiness in her urine and pain in her loins. In 1904 she was in the Bristol Royal Infirmary for four days for constipation. Two years ago she had extreme pain on passing her urine, which was relieved by treatment. She has had four or five attacks similar to this with pain in her loins. About three weeks before admission she noticed that her urine was cloudy, and had pain on micturition. She then suffered with a good deal of vomiting, and for two or three days could keep no food down. For four days she had felt a marked feeling of fatigue, and three days before admission she had a rigor, followed by another one the day before she was admitted. On admission her tongue was clean, but she looked rather ill. At 7 p.m. she had a rigor, the temperature going up to 105° F.

Nothing abnormal was found in heart or lungs. The abdomen was rather full, and she would not relax it. The hepatic dulness was from the fifth rib to one inch above the costal margin. The spleen was not felt. She was tender in both loins on bimanual examination; she also had some tenderness in the right iliac fossa on deep pressure. The urine was of low sp. gr., 1004, acid, with a cloud of albumen and a good deal of pus. White cells during rigor, 24,000, of which

89 per cent. were polymorphonuclears.
5 per cent. were lymphocytes.
3.5 per cent. were large lymphocytes.
2.5 per cent. were transitionals.

The following day she was put on urotropin (gr. x t.d.s.). The temperature in the evening was up to 104.2, but she had no rigor. The next day the tenderness in the flanks was much better, and the temperature rapidly falling. The pathological report received the following day described the chief organism found in the urine as "an atypical colon bacillus." Improvement continued, the albumen and pus became very much less in quantity. Four days later there was no albumen in the urine and only a few pus corpuscles seen under the microscope. A fortnight after admission atypical B.C.C. were still found in the urine. On October 29th the patient was again put on urotropin (gr. x t.d.s.). In spite of this the temperature went up and pain and tenderness again appeared in the right loin. With this recurrence there were no rigors. On November 5th she was put on helmitol (gr. v t.d.s.). The temperature became normal, and the pus diminished to a slight trace. On November 10th vaccine treatment was begun and continued till December 14th, the first injection being of 12 millions and the last 250 millions. There was still a very slight trace of albumen and pus in the urine when she was discharged.

It is well known that a bacilluria due to B. Coli may occur without giving rise to such alarming symptoms as those just described, and indeed that it is frequently present without giving rise to any symptoms. It occurs not infrequently after the specific fevers, especially typhoid, scarlet fever, measles and diphtheria, in children. In children also it appears to be one of the causes of nocturnal incontinence, the urine in some of these cases being turbid from the number of the organisms present. It is interesting to observe that these cases are often those associated with thread-worms.

I have ventured to bring these cases forward, as the cause of the symptoms may be overlooked in the absence of urinary symptoms, unless the possible presence of B. Coli bacilluria be borne in mind. Prominent among the symptoms which should lead to such a search in any given case of unexplained fever are—

(1) Multiple rigors.
(2) Markedly oscillatory temperature.
(3) Retching and vomiting.
(4) Relapses.
(5) Splenic enlargement.
(6) Abdominal tenderness and subcostal pain.
(7) Leucocytosis.

### HYPNOTISM.[1]

BY

HUGH P. COSTOBADIE, M.R.C.S., L.R.C.P.

## Figures and Tables

**Case I. f1:**
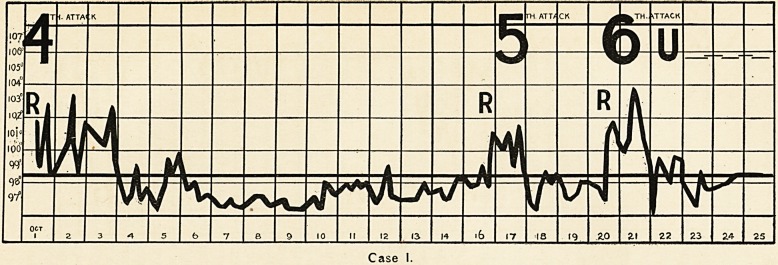


**Case IV. f2:**
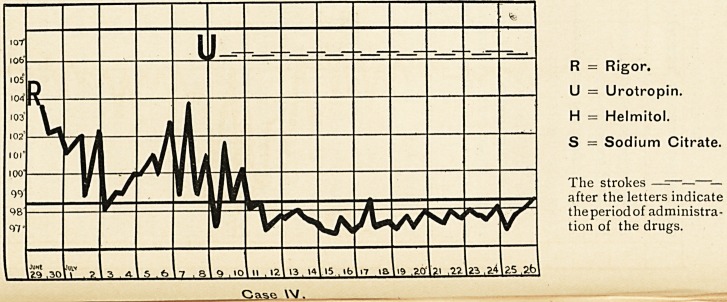


**Case V. f3:**
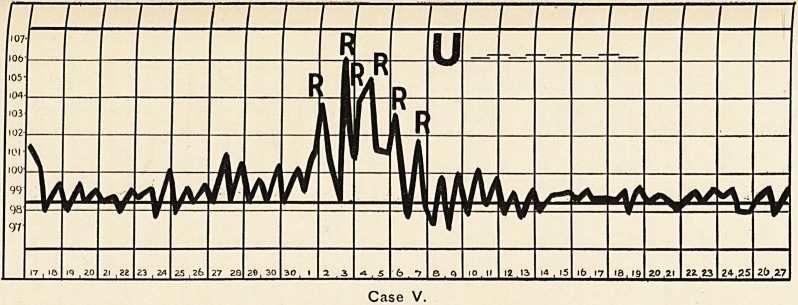


**Case VII. f4:**